# Late presentation of *RPE65* retinopathy in three siblings

**DOI:** 10.1007/s10633-019-09745-z

**Published:** 2020-01-10

**Authors:** Moustafa Magliyah, Amjad Ameen Saifaldein, Patrik Schatz

**Affiliations:** 1 Vitreoretinal Division, King Khaled Eye Specialist Hospital, Riyadh, Saudi Arabia; 2 Ophthalmology Department, Prince Mohammed Medical City, Aljouf, Saudi Arabia; 3 Ophthalmology Department, King Faisal Medical Complex, Taif, Saudi Arabia; 4 Department of Ophthalmology, Clinical Sciences, Skane University Hospital, Lund University, Lund, Sweden

**Keywords:** *RPE65*, Gene therapy, Multimodal retinal imaging

## Abstract

**Purpose:**

Gene therapy for *RPE65* retinopathy has been recently approved. The purpose of this study was to assess retinal structure and function in 3 siblings presenting with late-stage *RPE65* retinopathy and to assess the unmet need for such therapy in Saudi Arabia.

**Methods:**

Search of the retinal dystrophy registry at King Khaled Eye Specialist Hospital and clinical examination including multimodal retinal imaging, full-field electroretinography (ERG), dark adapted full-field stimulus sensitivity thresholds, and molecular genetic testing in 3 patients.

**Results:**

Nine (9) patients were identified with biallelic *RPE65* mutations, corresponding to a prevalence rate of 9/187 = 5% among early onset retinal dystrophies. Of these, 3 siblings (2 male and 1 female) with *RPE65* retinopathy were assessed in detail, because of an unusual, late presentation. They were all over 30 years old at the time of their most recent visits and had non-recordable ERGs. The 2 male siblings presented with poor vision and paracentral loss of the inner segment ellipsoid (ISe) and focal attenuation of the outer nuclear layer (ONL) in the macula. On the other hand, the female sibling presented with 20/100 vision with preserved foveal ISe and intact ONL throughout the macula and significantly lower light sensitivity thresholds compared to her male siblings. A homozygous missense p.Arg91Trp mutation in *RPE65* was identified in all. All patients were eligible for gene therapy, demonstrating a central retinal thickness of more than 100 microns on repeated examinations.

**Conclusions:**

*RPE65* retinopathy seems to be relatively common on the Arabian peninsula, and in addition it may be underdiagnosed. To the best of our knowledge, this is the first detailed presentation, including multimodal retinal imaging and electrophysiological assessment, of such patients from this region. Patients with late presentation of *RPE65* retinopathy may be eligible for gene therapy, in terms of remaining retinal function and structural preservation. The therapeutic window of such therapy remains to be determined.

**Electronic supplementary material:**

The online version of this article (10.1007/s10633-019-09745-z) contains supplementary material, which is available to authorized users.

## Introduction

Leber’s congenital amaurosis (LCA) is a group of congenital retinal dystrophies which usually present before 6 months of age with severe visual impairment and nystagmus [1–5]. Inheritance is autosomal recessive; however, dominant inheritance has been reported in cases with *CRX* and *IMPDH1* mutations [6–11].

Mutations in several genes have been shown to cause recessive LCA (reviewed in https://www.ncbi.nlm.nih.gov/books/NBK1298/). Recessive *RPE65* retinopathy has traditionally been grouped with LCA; however, it differs because useful vision and central retinal structure may be preserved for several years [12]. Together with slow progression, this makes it a potential candidate for gene therapy [13]. *RPE65* encodes the isomerohydrolase of the visual cycle, and dysfunction of this enzyme leads to an insufficient regeneration of the chromophore linked to opsin in the photoreceptors. This also leads to a slowly progressive degeneration of the retina. Typically the full-field ERG is non-recordable even at the time of initial diagnosis and therefore other form of assessment of progression, or of therapeutic response after gene replacement therapy, is needed, such as visual fields, visual acuity, full-field sensitivity testing (FST) and multimodal retinal imaging, including assessment of preservation of retinal layers and retinal thickness.

In this study, we describe the clinical, electrophysiological and molecular genetic findings in 3 siblings who presented with late-stage *RPE65* retinopathy, in light of the recently approved gene therapy for this condition. To the best of our knowledge, such assessment including detailed analysis of retinal structure and function has not been described for this disease in the Arabian peninsula. The recently approved gene replacement therapy is expensive and resource demanding and therefore may require significant planning and coordination. Thus in addition, we estimate the prevalence of this condition in the region, in preparation for future gene therapy.

## Methods

Informed consent was obtained, and the study was approved by an institutional review board at King Khaled Eye Specialist Hospital, which is a nation-wide tertiary referral centre, and at times also accepts referred patients from neighbouring countries. Ophthalmic examination including multimodal retinal imaging and full-field electroretinography (ERG) was done as described by us and others previously [14, 15]. Goldman visual fields were performed using objects V4 and III4.

ERG (Nicolet Biomedical Instruments, Madison, Wisconsin, USA) was obtained as follows, in dark adapted and light adapted state according to ISCEV standards [15], with a few modifications as follows. Dark adaptation was performed for 30 min, and dilatation of the pupils was obtained with topical cyclopentolate 1% and metaoxedrine 2.5%. After topical anaesthesia, a Burian Allen bipolar contact lens was placed on the cornea and a ground electrode was applied to the forehead. Responses were obtained stimulating with single full-field flash (30 ms) with blue light (0.81 cd s/m^2^: rod response) and with white light (10.02 cd s/m^2^: combined rod–cone response). In addition, the dark adapted cone response was measured after stimulation with dim red light (3.93 cd s/m^2^). Photopic responses were obtained with a background illumination of 3.4 cd s/m^2^ in order to saturate the rods.

Dark adapted full-field stimulus thresholds (FST) were assessed using white light and the Espion ColorDome™ system (Diagnosys LLC). FST measures the light sensitivity over the whole visual field and is therefore not affected by nystagmus. A meaningful change has been suggested as 10 dB or 1 log. Results were presented as log candela seconds per square meter (log cd s/m^2^).

A targeted next-generation sequencing (NGS) was performed using two panels in two different laboratories; a retinal dystrophy panel at molecular genetics laboratory at King Faisal Specialist Hospital (KFSH) [16], and a LCA panel in the Bioscientia Human Genetics laboratory (Bioscientia, Ingelheim, Germany) which includes *GUCY2D*, *RPE65*, *SPATA7*, *AIPL1*, *LCA5*, *RPGRIP1*, *CRX*, *CRB1*, *CEP290*, *IMPDH1*, *RD3*, *RDH12*, *LRAT*, *MERTK* and *TULP1*.

Finally, we searched a newly established retinal dystrophy registry at King Khaled Eye Specialist Hospital, which includes most patients seen in specially designed “retinal dystrophy clinics” since 2014, at the time of which electronical medical records were implemented in the hospital, for “RPE65”, in order to estimate the prevalence of *RPE65* retinopathy in the region.

## Results

The retinal dystrophy registry at this time included a total of 789 patients. Of these, 187 patients for whom NGS had been carried out were diagnosed with early onset retinal dystrophy. Nine (9) of these patients had biallelic *RPE65* mutations, arriving at an estimated prevalence rate of 9/187 = 5% among early onset retinal dystrophies.

Three of the affected patients, 3 siblings, had an atypical late presentation of the disease, while the other 6 patients had all been diagnosed before reaching 10 years of age. The 3 patients were further examined in detail, because of the unusual late presentation, with the aim of assessing the extent, if any, of remaining retinal function. The patients were belonging to a family of 8 siblings (3 brothers and 5 sisters) with a positive history of parental consanguinity. All three, 2 male and 1 female (patients 1, 2 and 3), aged 32, 36 years and 34 years old, respectively, at most recent follow-up, were homozygous for the c.271C > T (p.Arg91Trp) mutation in *RPE65*. The mutations were confirmed by 2 independent molecular genetic institutions (KFSH for patients 1 and 3 and Bioscientia for patient 2); however, other family members were not available for mutation segregation analysis or clinical examination.

**Fig. 1:**
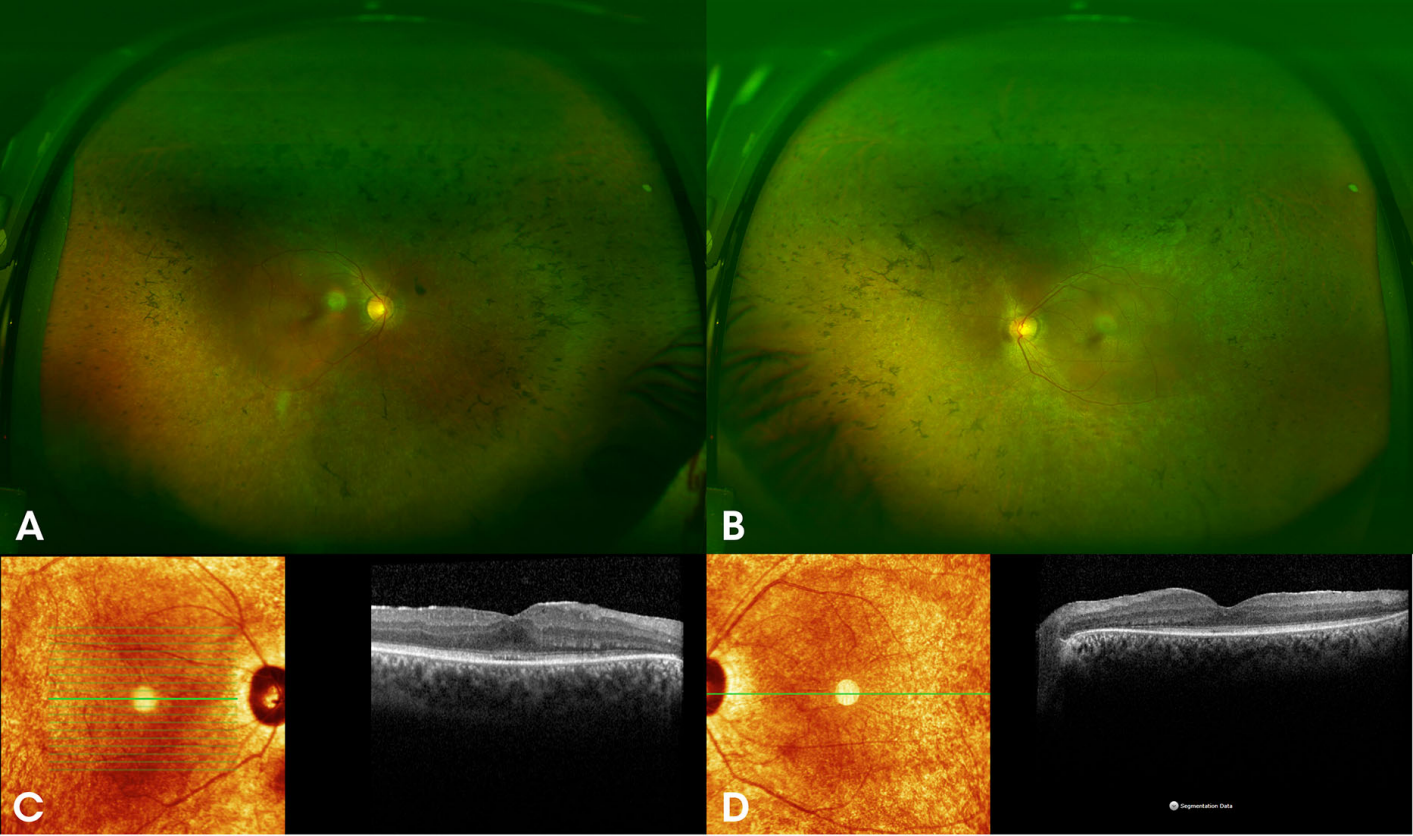
**A** and **B** colour fundus photographs of both eyes of a 32-year-old male patient (patient 1) with the homozygous c.271C > T (p.Arg91Trp) mutation in *RPE65*, showing peripheral pigment depositions (bone spicules), attenuated blood vessels and oval shaped hyperopic discs with temporal pallor. Spectral domain optical coherence tomography (SD-OCT) of both eyes **C** and **D** shows paracentral loss of the inner segment ellipsoid (ISe) and focal attenuation of the outer nuclear layer (ONL)

**Fig. 2:**
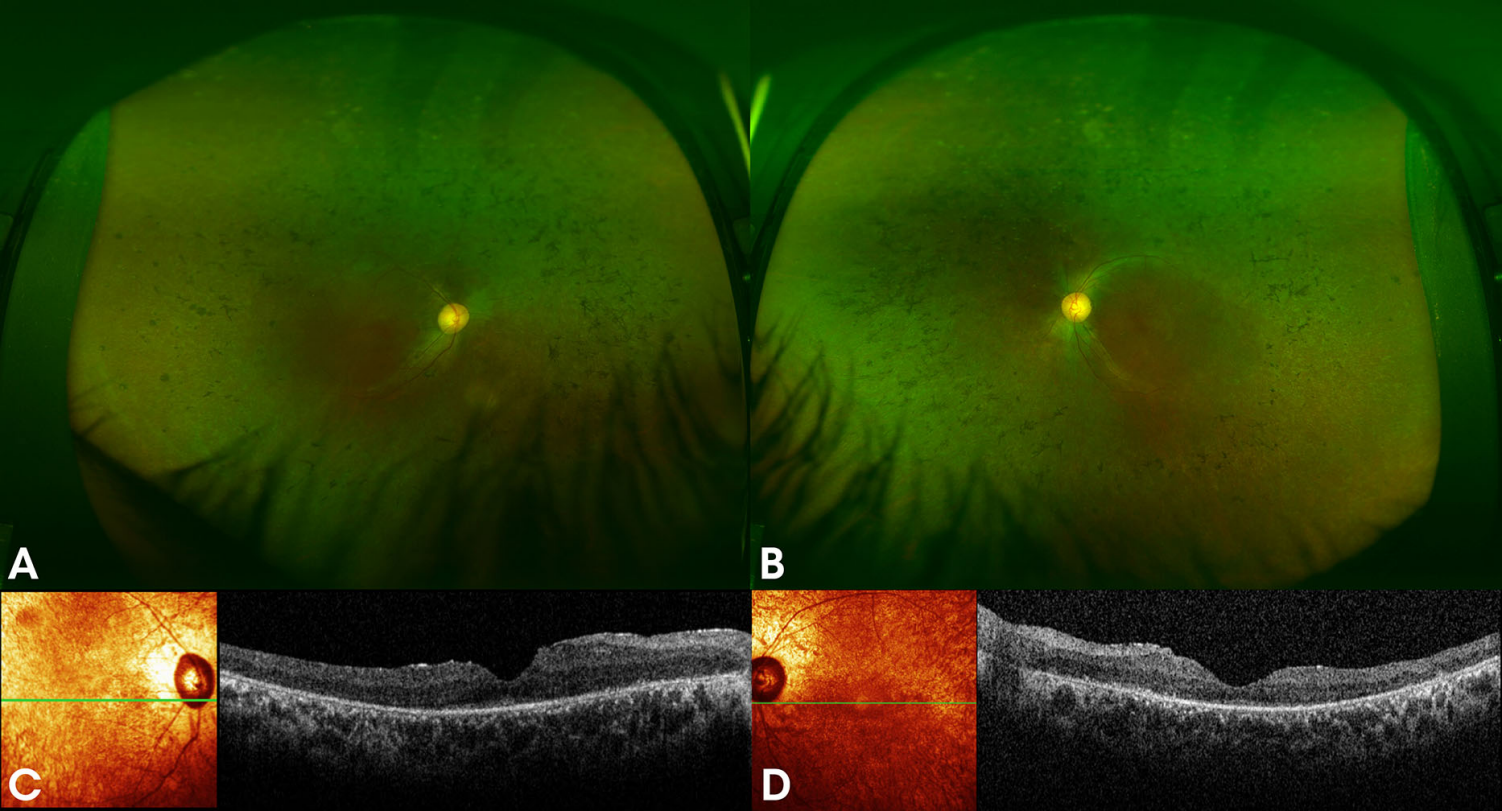
**A** and **B** colour fundus photographs of both eyes of a 36-year-old male (patient 2), sibling of patient 1, with the homozygous c.271C > T (p.Arg91Trp) mutation in *RPE65* showing bone spicules, attenuated blood vessels and oval shaped hyperopic discs with temporal pallor. Spectral domain optical coherence tomography (SD-OCT) of both eyes **C** and **D** shows paracentral loss of the inner segment ellipsoid (ISe) and attenuation of the outer nuclear layer (ONL)

**Fig. 3:**
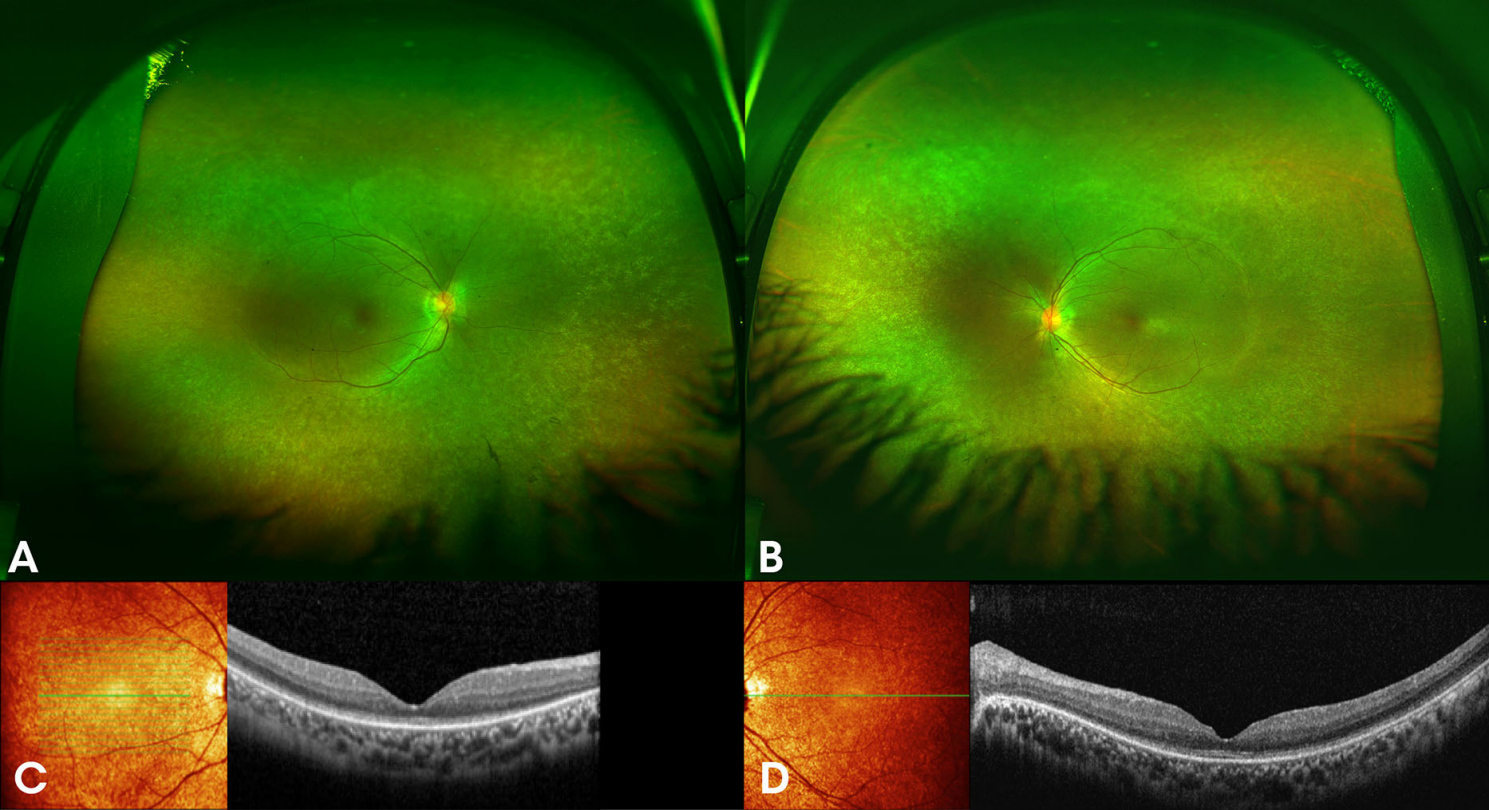
**A** and **B** colour fundus photographs of both eyes of a 34-year-old female (patient 3), a sibling of previous two patients, with the homozygous c.271C > T (p.Arg91Trp) mutation in *RPE65,* showing peripheral depigmentation, hyperopic oval shaped discs and attenuated vessels. Spectral domain optical coherence tomography (SD-OCT) of both eyes **C** and **D** shows paracentral loss of the inner segment ellipsoid (ISe) and relatively intact outer nuclear layer (ONL) throughout the macula

**Fig. 4:**
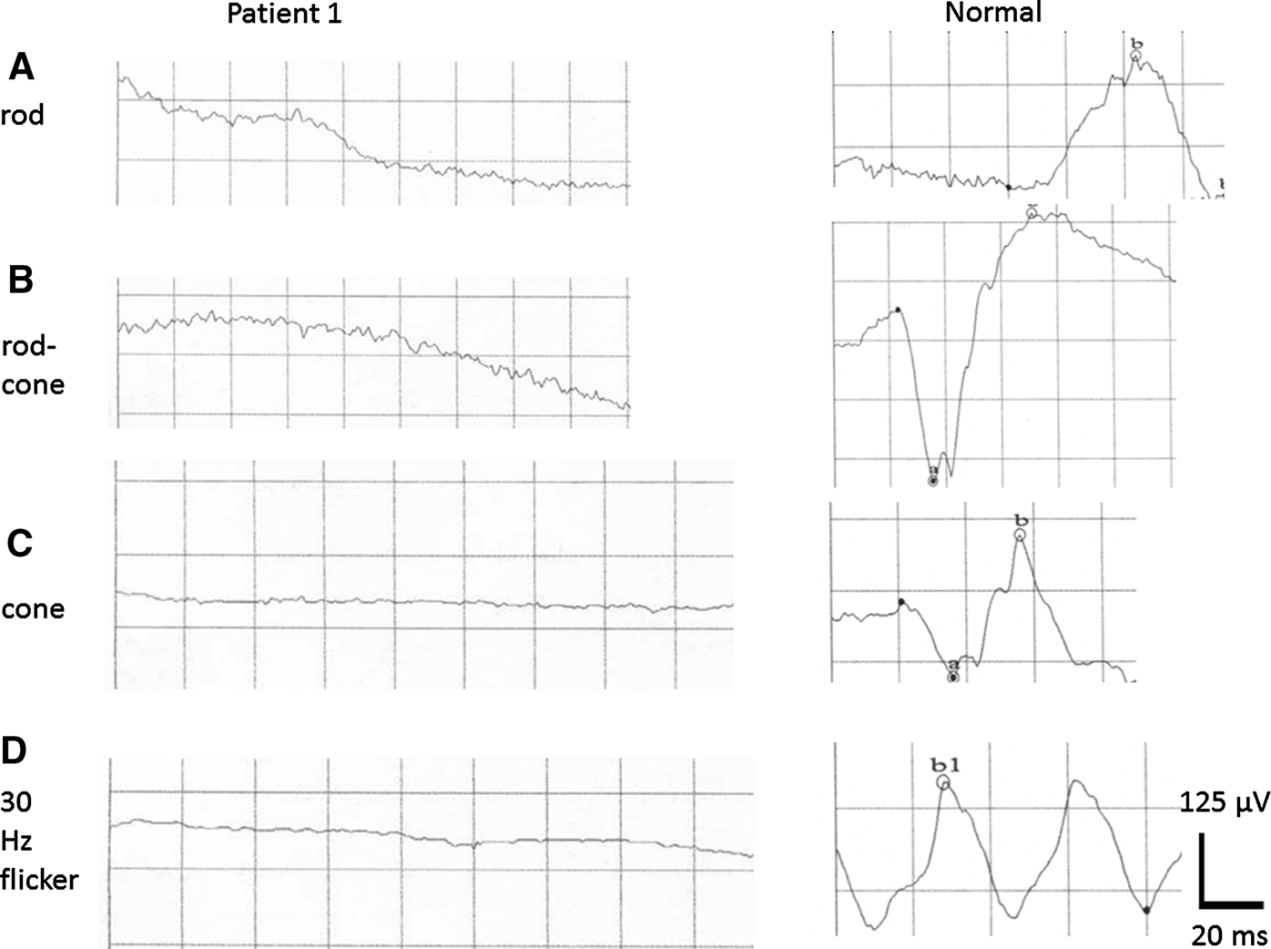
Full-field electroretinogram (ERG) in Patient 1 with *RPE65* retinopathy, at the age of 30 years, shows no recordable responses. A normal ERG is shown as a reference in the right column

All 3 had experienced nystagmus and night blindness since early childhood, with gradual and progressive visual loss. They presented 4 years ago with best corrected visual acuity (BCVA) of 20/400 in both eyes of patients 1 and 2, and 20/80 in patient 3. Patients 1 and 2 showed prominent peripheral retinal pigmentation, with less peripheral changes in patient 3. Central macular retinal layers showed signs of paracentral loss of the inner segment ellipsoid (ISe) and outer nuclear layer (ONL) compromise, more severe in patients 1 and 2, whereas the ONL was preserved throughout the macula in patient 3 (Figs. 1, 2, 3). ERG was non-recordable in all (Fig. 4 demonstrates the ERG obtained from Patient 1, and Supplemental document 1 demonstrates the ERGs of Patients 2 and 3). After 4 years of follow-up, BCVA was reduced to light perception (LP) in both eyes of the male patients and to 20/100 in the female patient.

In patient 3, an ETDRS OCT map was possible in the right eye on 2 separate occasions, separated by 30 months, showing an apparent decline of the central subfield thickness from 252 to 196 µm, while the visual acuity remained stable during the same period, ranging between 20/100 and 20/80 during a total of 7 visits. Retinal thickness was well above 100 µm in all subfields, in both eyes. In the other 2 patients, a quantitative approach over time including analysis of potential changes of retinal thickness was not possible because of insufficient fixation during obtaining OCT images in most of the visits, due to nystagmus; however, available thickness measurements from single-line horizontal transfoveal scans demonstrated a retinal thickness well above 100 µm along the scanned lines.

Visual fields were severely constricted to less than 10 degrees with the largest object in Patients 1 and 2, while Patient 3 had constricted fields to less than 10 degrees with the III4 object and less than 60 degrees horizontally and less than 50 degrees vertically with the largest V4 object. Mean (of right and left eyes for each patient) FST thresholds were 2.7, 1.9 and − 1.8 log cd s/m^2^, for Patients 1, 2 and 3, respectively.

Fundus autofluorescence demonstrated a generalized loss of autofluorescence, where the discs appeared relatively bright on a dark background; however, a trace of preserved autofluorescence could be noticed within the vascular arcades, compared to the periphery, especially in Patient 3 (Supplemental document 2).

## Discussion

We describe a late presentation of *RPE65* retinopathy in 3 siblings with the homozygous p.Arg91Trp mutation. King Khaled Eye Specialist Hospital is a major tertiary referral centre on the Arabian peninsula, and we have so far identified 9 patients with *RPE65* retinopathy during our experience of more than 5 years in the hospital. This estimates the minimum prevalence of *RPE65* retinopathy among retinal dystrophies at 5% which is higher by a factor of 5 compared to a previous study in the US population [17]. This may be due to a high rate of consanguineous marriage. Furthermore, the condition may be underrecognized and underdiagnosed due to limited availability to perform genetics testing, cost and failure to recognize the phenotype. However with the recently approved gene therapy for this condition, it is likely that the awareness of this condition, among ophthalmologists and patients alike, will increase, which may lead to identification of significantly greater number of patients.

We assessed 3 of these patients further, carrying the p.Arg91Trp mutation, which has been described previously in *RPE65* retinopathy [18–20]. Table 1 shows the phenotypic features of all patients with p.Arg91Trp mutation in *RPE65* including the three siblings in this study. Features in common for these patients include nystagmus, hyperopia, and non-recordable ERGs. Ambulatory vision was maintained in the female sibling (20/100 OU), similar to two patients described by Thompson et al. [20]. This could be due to a preserved RPE65 activity which can produce low amounts of 11-cis retinal in patients with the missense p.Arg91Trp mutation, allowing for better cone and rod function compared to patients with *RPE65* null mutations, as suggested in experimental work with mice with corresponding knock-in mutation, compared to *RPE65* null mice [21]. This may also account for some degree of preservation of fundus autofluorescence (Supplemental document 2). On the other hand, the 2 male patients in this study, similar to one of the patients described by Thompson et al., had poorer vision, which was noted also in the patient described by Habibi et al. [18] and was correlated with foveal atrophy on SD-OCT. The integrity of the ONL is a prominent indicator of photoreceptors function and was advised to be included in the evaluation for gene therapy [22].

**Table 1:** Phenotypic features of patients with the p.Arg91Trp mutation in the *RPE65* gene

Study	BCVA	Refraction	Fundus	OCT	ERG
Magliyah et al. this article	LP OU Nystagmus Age 36 years	+ 1 − 1.00X180 OU	Diffuse bone spicules, attenuated vessels, oval shaped hyperopic disc with temporal pallor	Paracentral loss of ISe, attenuation of ONL	Non-recordable
	LP OU Nystagmus Age 32 years	+ 3.50 − 1.00X90 OU	Diffuse bone spicules, attenuated vessels, oval shaped hyperopic disc with temporal pallor	Paracentral loss of ISe, attenuation of ONL	Non-recordable
	20/100 20/100 Nystagmus Age 34 years	+ 1.75 − 1.00X90 OU	Peripheral depigmentation, hyperopic oval shaped disc, attenuated vessels	Paracentral loss of ISe, intact ONL	Non-recordable
Habibi et al. [18]	< 20/200 Age 14 years	N/A	Depigmented deposits in the mid periphery, Mild optic atrophy, Narrowing of the vessels	Macular atrophy	
Li et al. [19]	Nystagmus Age N/A	+ 3.00 + 1.00X90 OU	Peripheral depigmentation, oval discs, mild optic atrophy and vascular attenuation	N/A	Non-recordable
	Nystagmus Age N/A	+ 1.50 + 1.00X90 OU	Minimal peripheral pigment changes; oval hyperopic disc	N/A	Non-recordable
	Nystagmus Age N/A	+ 0.25 + 1.50X90 OU	Minimal peripheral pigmentary changes; hyperopic full discs	N/A	Non-recordable
Thompson et al. [20]	20/100 OU, Nystagmus Age 7 years	N/A	N/A	N/A	Non-recordable
	20/100 OU Nystagmus Age 6 years	N/A	N/A	N/A	Non-recordable
	LP OU Age 23 years	N/A	N/A	N/A	Non-recordable

*BCVA* best corrected visual acuity, *HM* hand motion, *LP* light perception, *OU* both eyes, *ONL* outer nuclear layer, *ISe* inner segment ellipsoid, *OCT* optical coherence tomography, *ERG* full-field electroretinography, *N/A* not analysed, *y* years

Recently, Russell et al. [23] conducted a phase 3 randomized controlled trial evaluating the efficacy and safety of gene therapy for patients with *RPE65* retinopathy, in which four patients were compound heterozygous for the Arg91Trp mutation (and the mutations IVS7 + 2T > A, Trp402Stop [2 patients] and Arg91Gln, respectively). Table 2 shows that for this subgroup of patients, the average improvement in BCVA was 10 letters, which was similar to the mean for all patients (9 letters), while the average of illuminance level difference for passing the multiluminance mobility test (MLMT, the primary outcome of the study) was slightly less than the illuminance level difference mean for all patients (Table 2) [23].

**Table 2:** Results of gene therapy of patients with the Arg91Trp and (in a compound heterozygous, biallelic mode) IVS7 + 2T > A, Trp402Stop, Trp402Stop, Arg91Gln mutations, respectively, from top to bottom, in *RPE65* in the voretigene neparvovec phase 3 gene therapy trial by Russel et al. [23]

Patient	Age	Gender	Average BCVA at baseline	Average BCVA after 1 year	No. of letters gained	Illuminance (lux) passing level at baseline	Illuminance (lux) passing level at 1 year	Change Score
1	6	Male	20/125	20/100	8	4	1	1
2	34	Female	20/800	20/400	13	125	50	1
3	44	Female	20/800	20/500	11	125	10	2
4	19	Female	20/400	20/250	8	125	250	− 1

The Illuminance passing level refers to the level at which the patients were able to perform and pass the multiluminance mobility test, which was the primary outcome parameter [23]

*BCVA* best corrected visual acuity

In this study, in spite of late presentation, all 3 patients with the homozygous Arg91Trp mutation in *RPE65* maintained a central retinal thickness of more than 100 microns and also fulfilled other eligibility criteria, including the level of visual acuity, for gene replacement therapy. On the other hand, there was variability regarding the stage of disease, as observed by, for example, visual fields and remaining autofluorescence, among the 3 patients. Patient 3 presented with the least advanced disease stage, based on these assessments. Furthermore, based on the FST results, Patient 3 might be the best candidate, because of relatively preserved dark adapted sensitivity thresholds, presenting results that imply a sensitivity in the upper range compared to other patients with “LCA” who were examined previously in the literature by this modality, albeit the thresholds were significantly elevated compared to normal subjects who were reported to have a mean of −4.3 log cd s/m^2^ [24]. Thus, gene replacement therapy should be primarily be considered for Patient 3 once available in Saudi Arabia. This illustrates that FST is more appropriate than ERG as a measure of eligibility for and therapeutic response to currently available gene augmentation therapy, where the gene and its viral vector are delivered by subretinal injection in a limited area of the central retina only, and thus any improvement of retinal function in such a limited area may not be detected using ERG. Thus, albeit the ERG is a very powerful tool and is essential in diagnosing retinal dystrophies, the method has a limited sensitivity, and needs to be complemented with other more sensitive measures when evaluating potential treatments for retinal dystrophies.

Limitations of this study include the few number of patients examined and lack of mutation segregation analysis and clinical examination of other family members. On the other hand, this is the first report of clinical findings including multimodal retinal imaging in late-stage *RPE65* retinopathy due to this specific homozygous mutation, confirming eligibility for gene therapy for some patients, even with late presentation. A further novelty with this study is that *RPE65* retinopathy may be relatively common in Saudi Arabia, which may necessitate mobilization of economical resources and further diagnostic and operational skill in ophthalmic health care in the region. The therapeutic window for such therapy remains to be determined.

## Electronic supplementary material

Below is the link to the electronic supplementary material.

Supplemental document 1 Full-field electroretinography in 2 of the patients with *RPE65* retinopathy (Patients 2–3) demonstrating no measurable responses. (PDF 238 kb)

Supplemental document 2 Fundus autofluorescence findings in 3 patients with biallelic *RPE65* mutations, demonstrating a general reduction of autofluorescence; thus the discs appear relatively bright. However, note that there is some remaining autofluorescence around the vascular arcades in Patient 3, which may be due to some residual enzyme activity with some remaining production of 11 cis retinal. (PDF 416 kb)
