# Supplementary figures and images for: Late presentation of RPE65 retinopathy in three siblings
Source: Doc Ophthalmol. 2020 Jan 10;140(3):289–97. doi: 10.1007/s10633-019-09745-z (PMC7205780; doi:10.1007/s10633-019-09745-z)

Patient 2

Patient 3

Normal

Rod

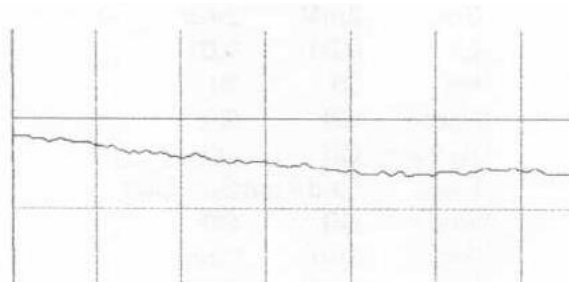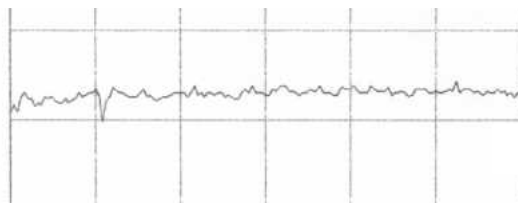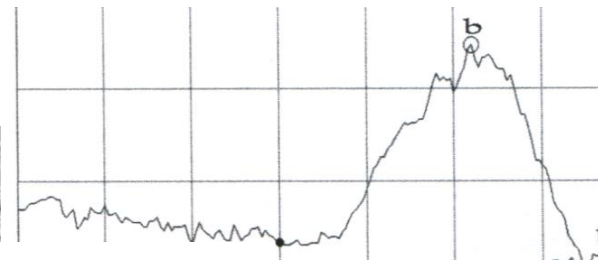

Rod-cone

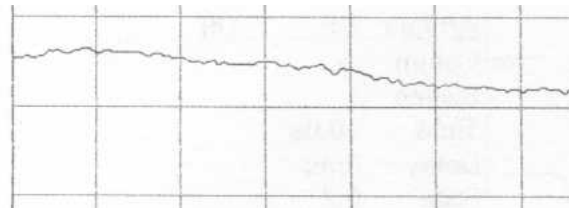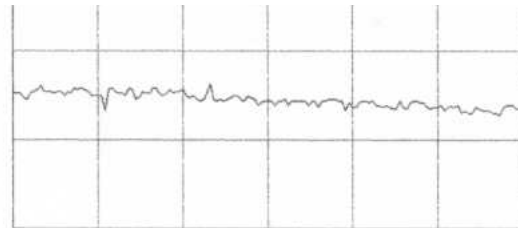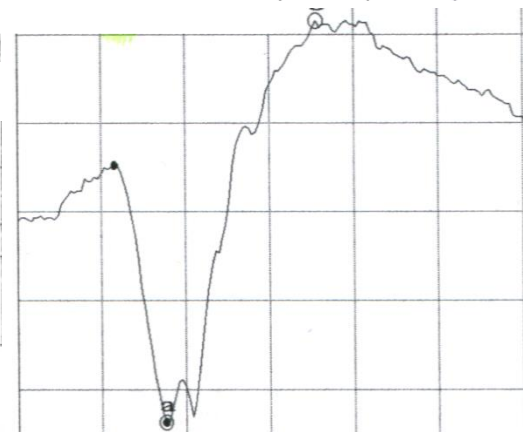

Cone

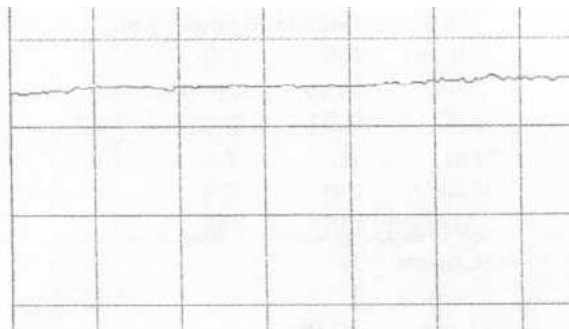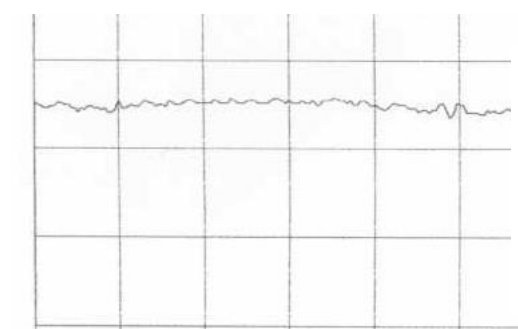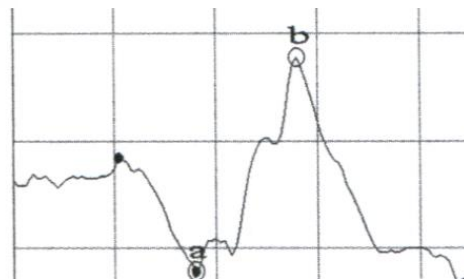

30 Hz flicker

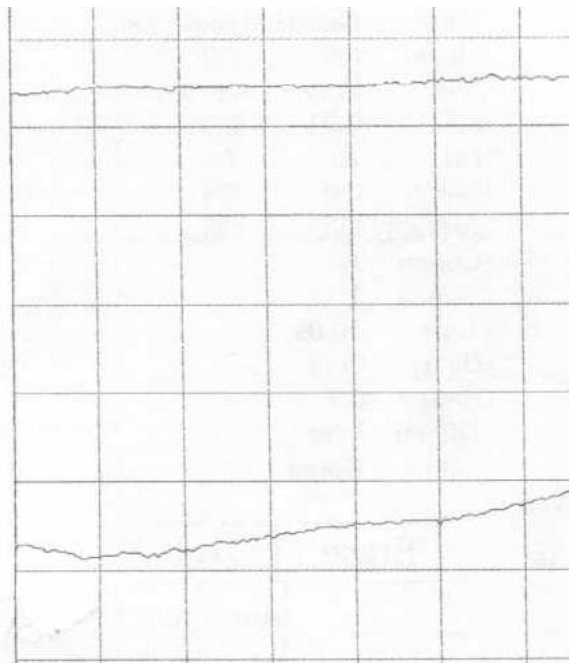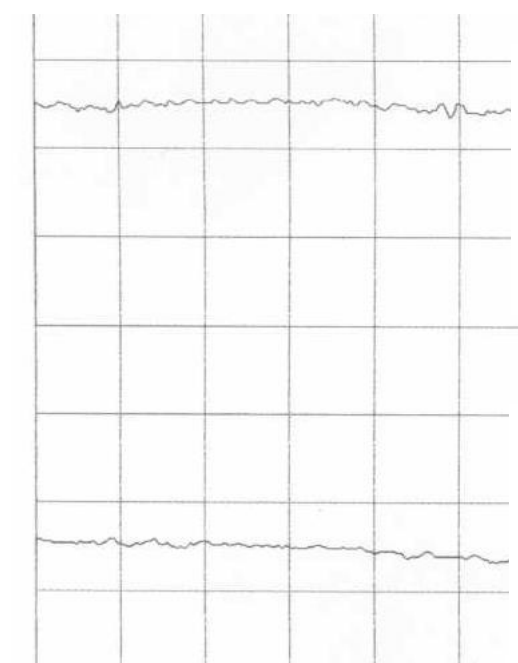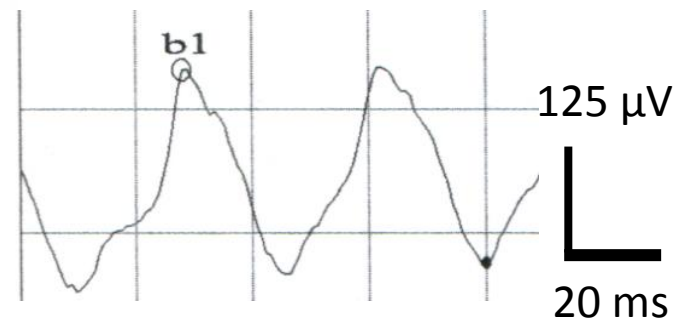

Supplement: Supplementary file 1 — Supplemental document 1 Full-field electroretinography in 2 of the patients with RPE65 retinopathy (Patients 2–3) demonstrating no measurable responses. (PDF 238 kb) [file 10633_2019_9745_MOESM1_ESM.pdf]

Patient 1

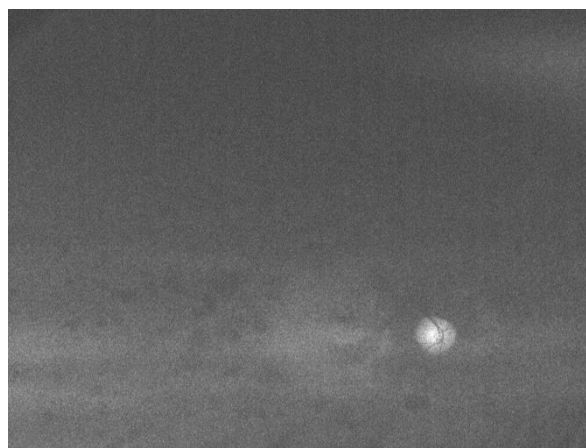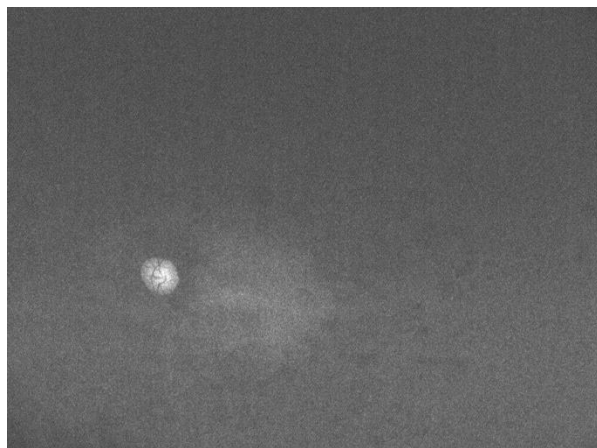

Patient 2

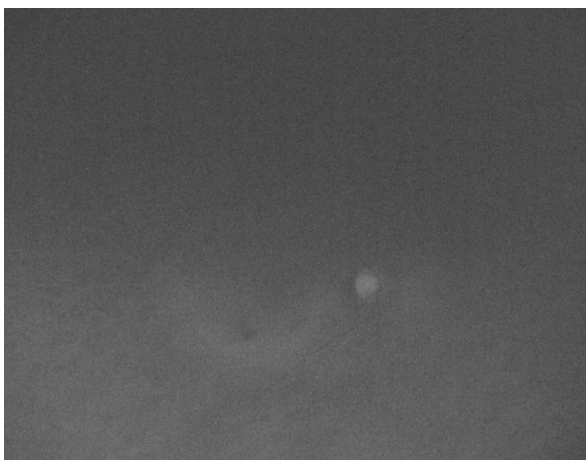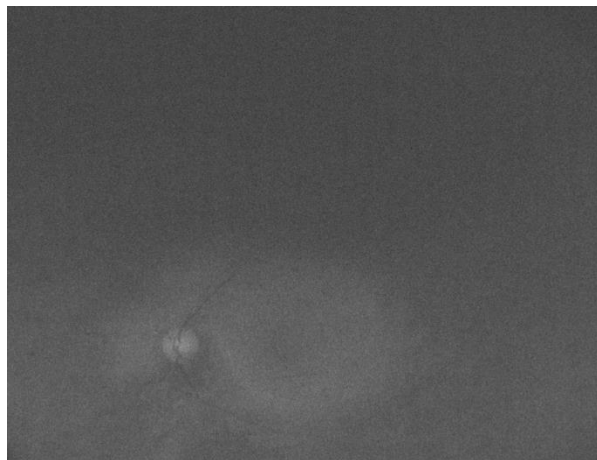

Patient 3

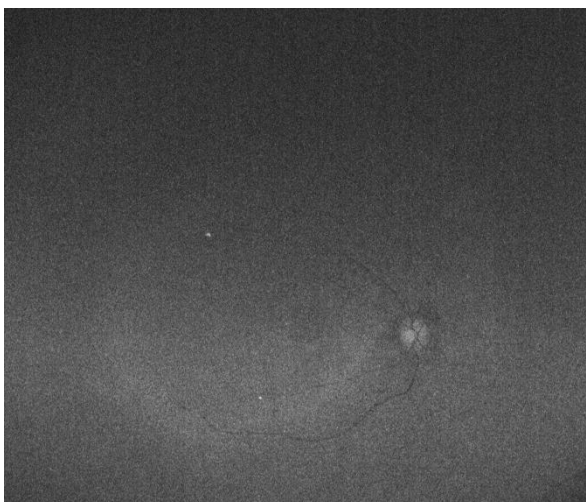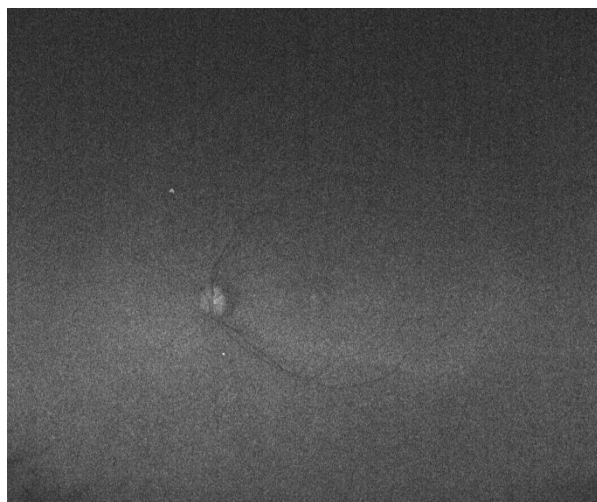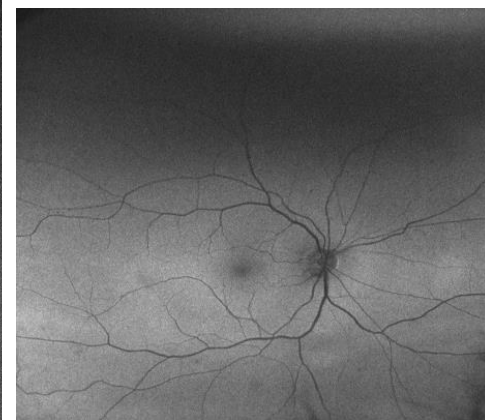

Normal

Supplement: Supplementary file 2 — Supplemental document 2 Fundus autofluorescence findings in 3 patients with biallelic RPE65 mutations, demonstrating a general reduction of autofluorescence; thus the discs appear relatively bright. However, note that there is some remaining autofluorescence around the vascular arcades in Patient 3, which may be due to some residual enzyme activity with some remaining production of 11 cis retinal. (PDF 416 kb) [file 10633_2019_9745_MOESM2_ESM.pdf]
